# Text Messaging to Extend School-Based Suicide Prevention: Pilot Randomized Controlled Trial

**DOI:** 10.2196/56407

**Published:** 2024-12-06

**Authors:** Anthony R Pisani, Peter A Wyman, Ian Cero, Caroline Kelberman, Kunali Gurditta, Emily Judd, Karen Schmeelk-Cone, David Mohr, David Goldston, Ashkan Ertefaie

**Affiliations:** 1 Department of Psychiatry University of Rochester Medical Center University of Rochester Rochester, NY United States; 2 Department of Pediatrics University of Rochester School of Medicine and Dentistry Rochester, NY United States; 3 Department of Internal Medicine University of Rochester School of Medicine and Dentistry Rochester, NY United States; 4 Department of Psychology Montclair State University Montclair, NJ United States; 5 Department of Preventive Medicine Feinberg School of Medicine Northwestern University Evanston, IL United States; 6 Department of Medical Social Sciences Feinberg School of Medicine Northwestern University Evanston, IL United States; 7 Department of Psychiatry and Behavioral Sciences Feinberg School of Medicine Northwestern University Evanston, IL United States; 8 Department of Psychiatry and Behavioral Sciences Duke University Durham, NC United States; 9 Department of Psychology and Neuroscience Duke University Durham, NC United States; 10 Department of Biostatistics and Computational Biology University of Rochester Rochester, NY United States

**Keywords:** suicide prevention, text messaging, self-violence, self-harm, suicidal behavior, randomized controlled trial, adolescent, teenager, student, school, United States, Text4Strength, help-seeking attitude, coping, awareness, depression, mood disorder, mental health

## Abstract

**Background:**

Suicide is the third-leading cause of death among US adolescents aged 10-19 years, and about 10% attempt suicide each year. School-based universal prevention may reduce youth suicidal behavior. Sources of Strength uses a peer leader network diffusion model to promote healthy norms across a school population. A key challenge within schoolwide programs is reaching a large and diverse array of students, especially those less engaged with their peers. Motivated by this challenge, we developed and field-tested Text4Strength—a program of automated text messages targeting help-seeking attitudes and norms, social coping resources, and emotion regulation skills.

**Objective:**

This study conducted a pilot randomized controlled trial of Text4Strength in 1 high school as an extension of an ongoing schoolwide program (Sources of Strength), to test its impact on targets that have the potential to reduce suicidal behavior.

**Methods:**

Students at an upstate New York high school (N=223) received 1-2 text messages per week for 9 weeks, targeting strategies for coping with difficult feelings and experiences through clarifying emotions and focusing on positive affect concepts, awareness, and strengthening of youth-adult relationships; and positive help-seeking norms, skills, and resources. Surveys were administered at baseline, immediately post intervention and 3 months after texting ended. We measured proximal intervention targets (methods of coping during stressful events, ability to make sense of their own emotions, feelings of powerlessness during emotion management and recovery, relations with trusted adults at school, and help-seeking behaviors), symptoms and suicide ideation, and student replies to messages.

**Results:**

No significant effects were observed for any outcome at either follow-up time point. Results showed that if there is a true (but undetected) intervention effect, it is small. Students with fewer friend nominations did not interact any more or less with the text messages. Exploratory moderation analyses observed no interaction between the intervention condition and the number of friends or baseline suicide ideation at any time point.

**Conclusions:**

In contrast to a promising previous field test, these results suggest that Text4Strength is unlikely to have impacted the outcomes of interest and that undetected moderate or large effects can be ruled out with high confidence. Although motivated by the need to reach more isolated students, students with fewer friends did not engage more or show a greater effect than other participants. This study was conducted in a single high school that was already implementing Sources of Strength, so the bar for showing a distinct effect from texting alone was high. Many further channels for reaching youth through private messaging remain unexplored. Alternative delivery systems should be investigated, such as embedding messaging in gaming chat systems and other media. More sophisticated systems drawing on chatbots may also achieve better outcomes.

**Trial Registration:**

ClinicalTrials.gov NCT03145363; https://clinicaltrials.gov/study/NCT03145363

## Introduction

Suicide is the third-leading cause of death among 10- to 19-year-olds in the United States, and about 10% of adolescents attempt suicide each year [1]. School-based universal prevention programs that address protective and risk factors across a population of students in adolescence [2-5] are emerging as viable ways to reduce youth suicide. Within such programs, help-seeking, youth-adult connectedness, and strategies for coping and regulating emotion are promising targets, given their documented association with reduced suicidal thoughts, behaviors, and antecedent risk factors [5-9]. However, a key challenge for school-based universal prevention programs remains—reaching a large and diverse array of students, especially those who are less engaged with school. The potential for disruptions to typical school schedules, large and small, also highlights the need for more flexible ways to reach students who do not rely on in-school contact alone.

Interventions that use automated text messaging have proliferated in recent years, and studies have shown efficacy and uptake with clinical populations of adolescents [10-13]. A randomized controlled trial (RCT) of an automated text–based intervention for adolescents who screened positive in an emergency department for depression and past-year violence was well received by patients and promising in terms of symptom improvement [14-16]. However, existing texting interventions have generally focused on clinical populations or treatment, with texting not aimed at youth in the general population. In contrast, we developed and previously field tested Text4Strength [17], a program of automated text messages that target help-seeking attitudes and norms, social coping resources [6], and emotion regulation skills to reinforce and extend school-based universal suicide prevention [7]. Text4Strength was specifically designed as an adjunctive and concurrent intervention to Sources of Strength [5], an established schoolwide suicide preventive intervention certified by the US National Registry of Evidence-based Programs and Practices [18].

The broader Sources of Strength intervention trains adolescent peer leaders to conduct SMS text messaging and other prevention activities aimed at promoting healthy coping and help-seeking norms and at strengthening youth-adult connections in a school network. Peers are trained and encouraged to deliver messages in a style and medium that will be most compelling for members of their friendship group. In an RCT conducted across 18 schools, the implementation of Sources of Strength correlated with increases in schoolwide health-seeking norms, with trained peer leaders 4 times more likely to refer suicidal friends to a trusted adult [5]. Results from an RCT with 40 schools testing the effect of Sources of Strength on self-reported suicide attempts are forthcoming [19].

Consistent with the core strategy of Sources of Strength, Text4Strength leveraged peer voices and strength-based testimonials [20], as well as other personalized SMS text messages, to reach students who are isolated or do not have strong friendship groups in school, as well as students with a higher risk of suicide, through this direct channel of communication. In addition to reinforcing the core concepts of Sources of Strength relating to norms about healthy coping resources, Text4Strength teaches specific skills for the self-regulation of emotion [7].

This study includes a pilot RCT of Text4Strength, delivered in 1 high school concurrently with Sources of Strength. To our knowledge, SMS text messaging has not yet been tested in an RCT to extend a universal school-based intervention nor to engage internal and social protective factors for suicide prevention. The purpose of this pilot RCT was thus to evaluate the effect of adding the SMS text messaging intervention as an additional component above and beyond the schoolwide Sources of Strength program. The key goal was to measure the magnitude and direction of the prevention effect on target outcomes, such as coping strategies, emotional self-regulation, youth-adult relationships, and help-seeking behaviors at the 3-month follow-up post intervention. We also sought to explore depression symptoms, anxiety symptoms, and suicide ideation, given their relationship to the primary intervention targets. However, our study was not powered to show significant differences in these exploratory outcomes.

## Methods

### Intervention and Adaptation

The Text4Strength intervention was adapted from an earlier version field tested with a sample of 43 ninth-grade students from 2 rural high schools and focused on the transition to high school [17]. In the field test, the students received 28 automated SMS text message sequences (16 with links to peer-leader videos) over 9 weeks (approximately twice per week). SMS text messages were sent outside school hours, and the content was not shaped by any school rules or policies. SMS text message sequences developed in collaboration with Sources of Strength peer leaders from 2 high schools [20] included lighthearted text-based games and activities, requests for advice, questions about students’ own experiences, and peer testimonial videos. Students who participated in this field test found Text4Strength to be appealing and useful. Almost all students replied to at least 1 SMS text message and read the messages even when they did not reply. Students engaged with text-based content more than video content, and reply rates were not related to levels of risk. Both struggling and relatively healthy adolescents were willing to engage with material aimed at building protective factors when presented in a positive, fun, and appealing way on their phones. Students gained awareness of their own feelings and learned new ways to handle upsetting situations, regardless of how many SMS text messages they replied to.

Based on lessons from the field test, we refined the design of the intervention. The SMS text message corpus was refined by (1) ensuring that the most critical prevention messaging was frontloaded in each sequence so students could determine whether the sequence was one they wished to explore further, (2) making SMS text messages more humorous and fun by including more games, and (3) personalizing the messages with students’ names and other tailoring. The refined intervention expanded upon the basic concepts that Sources of Strength presents through peer messaging and school-based prevention activities. The following targets were selected based on their relationship with suicide risk and attempts or related symptoms of depression and anxiety: (1) strategies for coping with difficult feelings and experiences through clarifying emotions and focusing on positive affect concepts (eg, positive events, gratitude, positive reappraisal, personal strengths, and making goals) [7,21-29]; (2) awareness and strengthening of youth-adult relationships [7,30-36]; and (3) positive help-seeking norms, skills, and resources [6,37].

The Sources of Strength program began in September and October with peer leader training and other activities. After 1-2 months of schoolwide activity to ensure broad student exposure to Sources of Strength concepts and peer leaders, students began receiving the revised corpus of SMS text messages at the frequency of 1-2 per week over the 9-week period.

A standardized protocol was in place to respond to any indications of suicidal ideation, whether spontaneously disclosed by participants or flagged through assessment responses. Trained research staff followed a decision tree to assess the nature and severity of suicidal thoughts, gathering information on whether thoughts were active or passive, any history of suicidal behavior, and current mood and agitation. If screening indicated a need for intervention, parents were contacted, and the school’s existing safety plans were activated. This could include referral to county crisis services or a local emergency department if warranted. Research staff remained available to assist schools and parents in determining appropriate resources and referrals.

### Participants

Participants were high-school students at a high school in upstate New York that was in its second year of implementing Sources of Strength. Parents or guardians of each student in the school (N=1029) received a letter home explaining the study and inviting participation. Immediately following this, we conducted informational meetings at the school for students and parents. Students were eligible to participate if they owned a cellphone, brought it to school, could connect to the internet, and could run apps on it. Of these, 246 (23.9%) students returned parent permission forms in response to initial and follow-up reminders from the school and research team. Of the 246 who returned forms, 223 students assented to participate, 220 students participated in the initial survey, and 206 students completed surveys at all time points. Tables 1 and 2 provide the demographic and covariate characteristics. The study sample did not differ from the school population by sex, race, or ethnicity. A larger proportion of ninth graders than other students participated.

**Table 1:** Demographic characteristics.

Demographics^a^		Control (n=109), n (%)	Intervention (n=111), n (%)	Total sample (n=220), n (%)	Difference at baseline? *P* value	
**Sex**						.69
	Male	51 (48.6)	49 (45.8)	100 (47.2)		
	Female	54 (51.4)	58 (54.2)	112 (52.8)		
**Grade**						.99
	9	44 (40.4)	44 (40)	88 (40.2)		
	10	24 (22)	23 (20.9)	47 (21.5)		
	11	21 (19.3)	23 (20.9)	44 (20.1)		
	12	20 (18.4)	20 (18.2)	40 (18.3)		
**Ethnicity**						.56
	Hispanic or Latino	2 (1.8)	1 (0.9)	3 (1.4)		
	Not Hispanic or Latino	107 (98.2)	108 (99.1)	215 (98.6)		
**Race**						.44
	Asian	0 (0)	2 (1.8)	2 (0.9)		
	Black or African American	5 (4.6)	4 (3.6)	9 (4.1)		
	American Indian	1 (0.9)	1 (0.9)	2 (0.9)		
	White	93 (85.3)	99 (89.2)	192 (87.3)		
	Multiracial	8 (7.3)	3 (2.7)	11 (5)		
	Other	2 (1.8)	1 (0.9)	3 (1.4)		
	Not reported	0 (0)	1 (0.9)	1 (0.5)		
**Peer leader**						.96
	Yes	13 (11.9)	13 (11.7)	26 (11.8)		
	No	96 (88.1)	98 (88.3)	194 (88.2)		

^a^8 students did not report sex, 1 did not report grade, 2 did not report ethnicity, and 1 did not report race.

**Table 2:** Covariate characteristics.

Covariate and time point					Control		Intervention		Total sample		*P* value	
												
**Construct and scale**												
	**Emotion regulation, mean (SD)**											
		**Limited ER^a^ Strategies**									.34	
			Baseline	1.81 (0.73)		1.77 (0.83)		1.79 (0.78)				
			T1^b^	1.84 (0.82)		1.82 (0.93)		1.83 (0.87)				
			T2^c^	1.88 (0.84)		1.84 (0.88)		1.86 (0.86)				
	**Clarity, mean (SD)**											
		**Lack of Emotional Clarity**									.03^d^	
			Baseline	2.32 (0.83)		2.14 (0.83)		2.23 (0.83)				
			T1	2.42 (0.77)		2.17 (0.76)		2.29 (0.77)				
			T2	2.51 (0.79)		2.23 (0.81)		2.36 (0.81)				
	**Coping behaviors, mean (SD)**											
		**Ways of Coping Checklist**									.75	
			Baseline	1.87 (0.63)		1.86 (0.61)		1.86 (0.62)				
			T1	1.97 (0.51)		1.96 (0.64)		1.96 (0.58)				
			T2	1.99 (0.62)		2.14 (0.62)		2.06 (0.63)				
	**Help-seeking norms, mean (SD)**											
		**Help-seeking from Adults at School**									.58	
			Baseline	3.00 (0.75)		2.97 (0.84)		2.98 (0.80)				
			T1	3.00 (0.72)		3.00 (0.72)		3.00 (0.72)				
			T2	3.08 (0.76)		3.10 (0.83)		3.09 (0.79)				
	**Norms about adult help, mean (SD)**											
		**Adult Help for Suicidal Youth**									.99	
			Baseline	3.30 (0.55)		3.29 (0.70)		3.29 (0.63)				
			T1	3.26 (0.58)		3.40 (0.66)		3.33 (0.63)				
			T2	3.32 (0.68)		3.38 (0.65)		3.35 (0.67)				
	**Trusted adults in family, school, and community, mean (SD)**											
		**Trusted Adults in School**									.86	
			Baseline	3.16 (0.70)		3.19 (0.80)		3.18 (0.75)				
			T1	3.25 (0.70)		3.31 (0.65)		3.28 (0.67)				
			T2	3.28 (0.81)		3.33 (0.73)		3.31 (0.77)				
		**Trusted Adults in Community**									.58	
			Baseline	3.07 (0.82)		3.14 (0.81)		3.10 (0.81)				
			T1	3.12 (0.81)		3.22 (0.80)		3.17 (0.80)				
			T2	3.25 (0.83)		3.30 (0.79)		3.28 (0.81)				
	**Adults to go to for help, n (%)**											.89
		**Yes**										
			Baseline	67 (60.9)		66 (60)		133 (60.5)				
		**No**										
			Baseline	42 (39.1)		45 (40)		87 (39.5)				
**Symptoms and scale, mean (SD)**												
	**Depressive symptoms**											
		**SMFQ^e^ Depression**									.09	
			Baseline	1.01 (2.32)		0.96 (2.48)		0.99 (2.39)				
			T1	1.11 (2.46)		1.03 (2.51)		1.07 (2.48)				
			T2	1.05 (2.66)		0.72 (2.31)		0.88 (2.49)				
	**Anxiety symptoms**											
		**PROMIS^f^ Anxiety**									.19	
			Baseline	2.10 (1.03)		1.97 (1.02)		2.04 (1.02)				
			T1	2.00 (1.03)		1.86 (0.98)		1.93 (1.00)				
			T2	2.11 (1.05)		1.99 (1.01)		2.04 (1.03)				
	**Suicide ideation symptoms**											
		**Suicide subscale from MFQ^g^**									.31	
			Baseline	0.63 (1.65)		0.56 (1.43)		0.60 (1.54)				
			T1	0.62 (1.46)		0.48 (1.47)		0.55 (1.47)				
			T2	0.66 (1.54)		0.53 (1.49)		0.59 (1.51)				

^a^ER: emotion regulation.

^b^T1: immediate follow-up.

^c^T2: follow-up 3 months after texting ended.

^d^Statistically significant *P* value.

^e^SMFQ: Short Mood and Feelings Questionnaire.

^f^PROMIS: Patient-Reported Outcomes Measurement Information System.

^g^MFQ: Mood and Feelings Questionnaire.

### Survey Procedure

Surveys were administered over the 2016-2017 school year before the start of the texting intervention (baseline; October to November 2016), immediately after 3 and a half months of texting (post intervention; March 2017), and again 3 months after texting ended (follow-up; May 2017). Surveys were administered by study staff members either in homeroom or a class and took about 15 minutes to complete.

### Measures

#### Overview

The baseline survey consisted of questions about descriptive information and intervention targets, as well as suicide ideation symptoms. The postintervention and follow-up surveys repeated measurement of intervention targets and suicide ideation symptoms. The follow-up survey also included feedback questions concerning the technical and content aspects of the SMS text messages received, as well as the perceived usefulness of the texts created by the research team. Suicide ideation symptom variables were exploratory because, while they are the ultimate targets of the intervention, the pilot RCT was not powered to test the impact on these outcomes. Alphas listed refer to those measured in baseline administration.

#### Baseline Descriptive Information

Students reported demographic information and mobile phone usage at school and named up to 7 close friends from a list of students at their school [38]. Students completed measures about socialization through cellphone and internet use [39], social acceptance and belonging (Integration with Peers; 4 items; α=.89) [40], positive and negative peer group behaviors [5,41], and general stress level (PROMIS [Patient-Reported Outcomes Measurement Information System]-Emotional Distress; 10 items; α=.92) [42,43]. In an effort to personalize SMS text messages, students were asked about their interests, such as favorite bands, TV shows, and movies.

#### Proximal Intervention Targets

Students completed measures on their methods of coping during stressful events (The Ways of Coping Checklist; 12 items; α=.88) [44], ability to make sense of their own emotions (Lack of Emotional Clarity subscale; 5 items; α=.78) [45], and feelings of powerlessness during emotion management and recovery (Limited Access to Strategies for Emotion Regulation subscale; 8 items; α=.87) [45]. Students also reported on their relationships with adults at school (Trusted Adults in School scale; could name up to 7 adults) [7] and help-seeking behaviors (Help-seeking from Adults at School scale; 4 items; α=.92, and Adult Help for Suicidal Youth scale; 4 items; α=.89) [37].

#### Symptoms and Suicide Ideation (Exploratory)

Students completed measures related to depressive symptoms (Short Mood and Feelings Questionnaire; 13 items; α=.91) [27,28] and anxiety symptoms (PROMIS-Pediatric Anxiety; 4 items; α=.88) [29,30]. Students who endorsed suicidal ideation on the depression survey were also asked if they were contemplating suicide (Short Mood and Feelings Questionnaire) [46,47].

#### Student Replies to Messages

Students randomized to the Text4Strength intervention group were sent a total of 36 sets of SMS text messages between baseline and T1 (immediate follow-up). For 30 (31 if high risk) of these sets, the first SMS text message invited a response that could lead to a branched sequence of 1-5 more messages depending on the students’ responses. In order to represent student interaction with messages, we measured their reply rate by calculating the proportion of message sequences replied to at least once. A total of 15 students declined to continue receiving texts (sent a stop message or asked to be removed) but are included in the denominator of the number of text sequences received.

### Randomization

Following the baseline survey, participants, stratified by sex and grade, were randomized into (1) the Text4Strength interactive text message condition or (2) the informative SMS text message control condition. All participants participated as usual in schoolwide Sources of Strength programming.

### Conditions

#### Text4Strength

In addition to schoolwide Sources of Strength programming, the Text4Strength intervention group received 2-5 personalized, interactive SMS text message sequences per week as an adjunct to the program. These messages were personalized based on information gathered during the baseline survey regarding “preferred name” and favorite band, TV show, and movie. The SMS text messages invited students to reply using keywords and short free-text replies. SMS text messages came from a library of strength-based peer quotations (reviewed for safety), psychoeducational interactions, and games designed to promote emotional skills and the use of resources. Students had 24-hour access to help information by texting “helpinfo” and could opt out of all future messages immediately by texting “stop.” Menu and keyword interactions are standard practices in SMS text messaging interventions, and our field test showed that most students intuitively understood this form of interaction [17,48-50]. Participants received periodic reminders that texts were automated (not monitored).

#### Information-Only Texts

In addition to schoolwide Sources of Strength programming, the control group received approximately 1 text message per week containing general Sources of Strength concepts (eg, “It’s important to think about what lifts us up when we go through tough times. Think about the protective factors in your culture, school, family & friends”; “Positive friends who help us make healthy decisions help us live healthy, full lives. Thank someone who’s a positive friend to you”; and “When someone you know is having serious problems, being a friend means getting an adult involved—not trying to handle it yourself. #trustedadults”). The control group had the same access to keywords for accessing information about getting help and for opting out of all messages.

### Analytic Strategy

The data for this pilot RCT were analyzed through an intention-to-treat analysis. To evaluate the effect of the intervention on each outcome, several univariate regression analyses were conducted—1 for each outcome at each of the 2 follow-up time points (ie, posttest and follow-up). Each outcome’s model included a dummy-coded treatment condition variable, as well as the baseline score for that specific outcome (ie, posttest score X=condition+baseline score X). The participants’ sex, grade, and peer leader status were also controlled for each model. This allowed us to evaluate the intervention’s potential effect on several outcomes while still accounting for any baseline differences initially present in those outcomes at the start of the study.

Standard power analysis techniques revealed this approach was adequately powered to detect a moderate effect size, with the power to detect Cohen *d*=0.40 estimated at 0.84. However, results also showed the study was weakly powered to detect a true effect smaller than that. For example, the power for a Cohen *d*=0.30 was estimated to be 0.60. Because the power to detect a small effect was weak, we also evaluated the study’s power to at least reject large effects and to bound a truly small intervention effect below a certain threshold [51]. Results of this second power analysis revealed that if the true effect of the intervention is approximately 0, our analysis models can rule out effect sizes larger than *d*=0.40 with power 0.91 and effect sizes larger than *d*=0.30 with power 0.72. All secondary tests were 1-tailed, as the goal was only to bound the true effect below a desirable intervention effectiveness threshold (eg, *d*=0.40). Furthermore, this approach adds to the clarity of study results by providing a positive confirmation of small-or-null effects rather than just the absence of a significant large effect [52]. Taken together, these analyses are thus adequately powered (1) to detect a “moderate” sized intervention effect and (2) in cases in which a significant moderate effect is not observed, to establish that the effect of the intervention is significantly less than “moderate” (ie, practically equivalent to 0).

Although not predicted a priori, we also conducted auxiliary analyses on multiple plausible intervention moderators assessed at baseline. These analyses were identical to the univariate regressions above, except that they also included a term for the baseline score of the moderator and the interaction between the moderator and the dummy-coded condition variable.

### Ethical Considerations

This study was reviewed and approved by the University of Rochester Research Subjects Review Board (STUDY00001008). Informed consent was obtained by sending a letter explaining the study to parents and guardians of each student at the school and conducting informational meetings for those interested in participating. Parent permission and student consent to participate were both required for participation in the study. Participant data were not anonymized but were deidentified after data collection was complete.

Students were paid US $5 for completing each of the follow-up surveys (US $10 in total). All study survey materials and recruitment procedures were approved by the University of Rochester Research Subjects Review Board. All survey data were collected using the University of Rochester’s secure REDCap (Research Electronic Data Capture; Vanderbilt University) system. Data were stored separately from identifying information on encrypted servers housed in a secure local data center. Access to data was restricted using role-based permissions. Usage data from SMS text message interactions were stored in a separate database using a HIPAA (Health Insurance Portability and Accountability Act)–compliant commercial SMS service with encrypted, dedicated servers. All data collection, storage, and handling procedures were in accordance with institutional policies and industry standards for protecting sensitive information.

## Results

**Figure 1:**
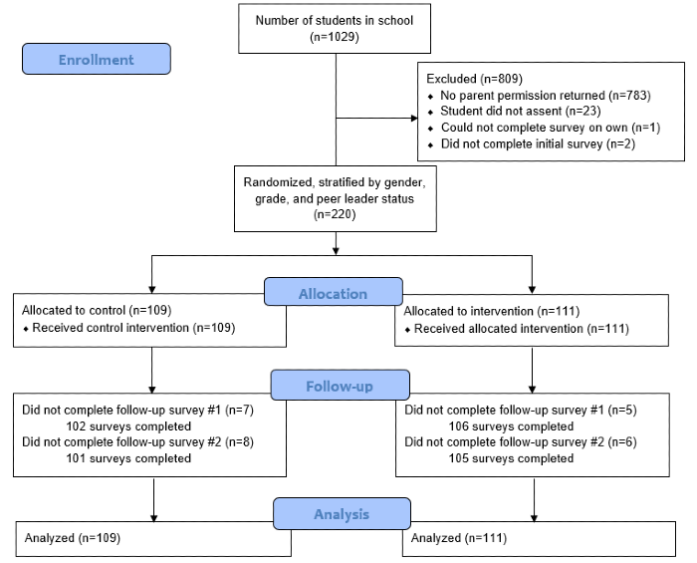
Text4Strength pilot randomized controlled trial CONSORT (Consolidated Standards of Reporting Trials) flow diagram.

**Figure 2:**
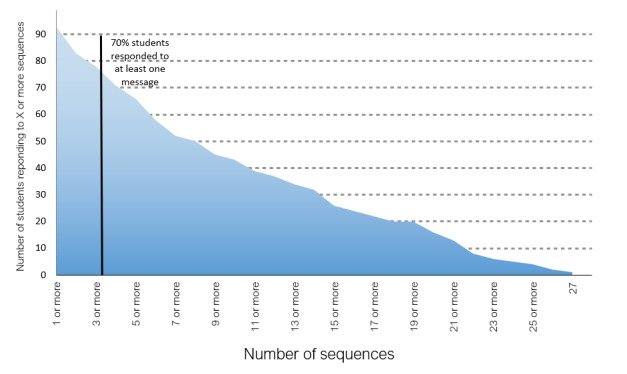
Number of sequences to which students responded.

Figure 1 shows a CONSORT (Consolidated Standards of Reporting Trials) diagram of the participants included in this study. As shown in Table 1 and Table 2, the groups were stratified so they would not differ by sex, age, or peer leader status. No group differences were found by race or ethnicity. Regarding mobile phone use, 80% (176/220) of students reported sending and receiving SMS text messages every day, with only 1.8% (4/220) responding “Never.” Fewer students, 57.3% (126/220), reported talking to people they knew on their cell phone every day, with 2.3% (5/220) responding “Never” and 18.5% (41/220) responding “Several times a week.” Furthermore, students consistently engaged with SMS text messages. As shown in Figure 2, a total of 92 (83.6%) out of 110 students replied to 1 or more SMS text messages, and 77 (70%) responded to 3 or more. The proportion responding to any given message that invited a reply ranged from 3.6% (4/110) to 87.3% (96/110), with students responding to a mean of 8.7 (SD 7.84) messages. Female students showed a trend for responding to a greater percentage of SMS text messages than male students (female: mean 33.4, SD 26.71; male: mean 24.5, SD 23.90; t_103_=1.80, *P*=.08). Student replies were appropriate, generally on point, and no safety concerns were detected.

As shown in Table 3, no significant intervention effects were observed for any outcome at any of the 2 postbaseline time points. Consistent with this finding, results also showed that if there is a true (but undetected) intervention effect, it is significantly weaker than *d*=0.40 for all variables, except possibly total depression. Taken together, these results suggest that the intervention is unlikely to have impacted the outcomes of interest and that undetected large effects can be ruled out with high confidence.

A similar pattern emerged in exploratory moderation analyses. Specifically, no interaction was observed between the intervention condition and the total number of friends at baseline for any outcome at any subsequent time point. Similarly, no interaction was observed between condition and baseline suicide risk for any outcome at any subsequent time point. Thus, the effect of the intervention does not appear to differ for any of the subgroups examined here.

In an additional set of auxiliary analyses, rates of reply to intervention SMS text messages were also examined. Results showed rates of reply were significantly positively associated with both posttest and follow-up anxiety scores, but with no other outcome variables (β=.011, SE=0.003, *P*=.002 in both cases). However, given the number of tests conducted in this investigation and the lack of significant effects of the intervention on anxiety scores, this finding is difficult to interpret and is likely a false positive.

**Table 3:** Effect of condition on study outcomes at posttest and follow-up (effect sizes were calculated after controlling for sex, grade, and peer leader status).

Outcome	Posttest		Follow-up	
	Cohen d	95% CI	Cohen d	95% CI
Anxiety	–0.01	–0.29 to 0.27	–0.01	–0.29 to 0.27
Emotional clarity	–0.02	–0.30 to 0.26	–0.02	–0.30 to 0.26
Help seeking acceptance	0.00	–0.28 to 0.28	0.00	–0.28 to 0.29
Suicidal ideation	–0.03	–0.31 to 0.26	–0.03	–0.31 to 0.26
Total depression	–0.15	–0.43 to 0.13	–0.16	–0.45 to 0.12
Limited access to coping strategies	0.00	–0.29 to 0.28	–0.01	–0.29 to 0.27
Trusted adults	–0.03	–0.31 to 0.25	–0.01	–0.30 to 0.27
Ways of coping	0.00	–0.28 to 0.28	0.02	–0.27 to 0.30

## Discussion

### Principal Findings

In contrast to a promising previous field test, these pilot RCT results suggest that it is unlikely that the intervention impacted the outcomes of interest. No significant effects were observed for any outcome at either follow-up time point, and undetected moderate or large effects can be ruled out with high confidence. Contrary to our expectation, students with fewer friends did not engage more or show a greater effect than other participants. However, there is still much to learn from this study.

Effective promotion in schools of protective factors for suicide risk requires that interventions reach and engage a diverse range of students with varying individual circumstances. To our knowledge, this study is the first to use SMS text messaging to extend a universal schoolwide intervention aimed to engage protective factors for suicide prevention. In the absence of a comprehensive “one-size-fits-all” intervention, the most promising approach is to use a variety of channels to engage students. Previous work has shown that participants engage with text-based interventions in proportion with their own readiness and internal motivation for what the intervention offers [53]. In our study, students received Text4Strength messages in conjunction with a schoolwide program, not because of a mental health need or their own motivation. We thus expected a wide range of motivation and engagement from our students. The literature also suggests that automated, technology-centric interventions have robust, albeit small, effect sizes [12]. However, we did not find such an effect in our pilot RCT of Text4Strength.

Follow-up analyses revealed that the null effects in this pilot are not merely a result of insufficient sample size or power and that any undetected effect could only have been very small (ie, practically insignificant). Furthermore, in post hoc analyses, we tested a number of carefully selected variables that reflect some of the factors motivating the development of this program. For example, at the outset, we were seeking ways to reach more isolated students who might not have direct contact with peer leaders but who might interact more readily with texted materials on their phones. The post hoc analyses were carried out on the assumption that it should be easier to show an effect for such groups than for the broader sample. But in no case did we find any significant effect. The results of the post hoc analyses also showed that this group did not interact any more with the texts than did other participants, a finding that mirrored our previous field test, in which no participant characteristic (distress, depression, anxiety symptoms, and coping support) was related to text reply rates. Another result that remained consistent across the field test and the present study is that engagement with the texts did not correlate with how useful a person found them. Just as in the field test, the number of texts replied to was not correlated with how useful the texts were found to be. Here, as elsewhere, the amount of engagement was not associated with a greater or lesser effect in relation to the target variables.

The null effects observed in this pilot RCT nevertheless provide an opportunity to reflect on why the addition of a texting element to the ongoing Sources of Strength program did not yield its intended effects. The high bar of trying to show an effect above and beyond that of the current evidence-based schoolwide intervention may well have presented an obstacle to identifying an effect arising from the texting alone, and we do not know what the effect of a texting intervention promoting peer norms would be in comparison with no intervention. This could be a useful question to follow up on since the cost and effort involved in implementing this intervention are so low.

Other possibilities can also usefully be considered. One of the motivations for reaching out directly to people’s phones and pockets is that the broader Sources of Strength intervention was directed at friendship circles, and some people have fewer or no friends. What we found here was that those people with less friends did not, in fact, engage more or show a greater effect than other participants. However, this intervention still placed a heavy emphasis on peer models, so the results of this study may suggest that we need an entirely different way of attracting people who are not very connected with their peers. For example, innovative work is currently taking place that seeks to reach youth through messaging in video gaming, taking mental health interventions out of the school friendship network, and into a preferred activity or subculture [54].

The technological system of prompts and responses used in this pilot was rudimentary but can be considered a proof of concept for future development. The system was primarily driven by keywords and had only a limited number of response options, with no user-initiated element or natural language processing capabilities. These elements have been incorporated successfully into other SMS text messaging interventions, for example, into a program for youth with asthma [55], and could feasibly be integrated with a revised version of the system used in this pilot [56-58]. In addition, our web-based procedure for coaching students to create positive peer testimonials was both feasible and useful, as demonstrated in our previous studies [17,20]. This technique could be adapted for use in other settings and interventions.

One important point to consider is that there were many variables across the student body that could have impacted the results, and these were not held constant in the study design in such a way that would enable us to pinpoint the source of effects across groups. Our post hoc analyses began to probe into possible variables of interest by looking for variation in people who were most distressed or engaged, but these results all came back null. However, there are many other variables that may potentially be relevant. For instance, one important variable that may be particularly useful to explore in the future is the timing of SMS text messaging and how that fits into the lives of participants. Timing of message delivery can have a large impact on the frequency of replies at a given time, but this has not yet been systematically varied. A valuable start point would be to ask participants about good times to text them and to include this data in the personalization of the automatic texting. While smart systems might be able to identify appropriate times with a high level of accuracy, in future iterations, we should aim at a minimum to establish preferred times of day for each individual. However, we should be cautious about thinking that an intervention like this is something that participants “complete” and that we should necessarily be aiming for as many responses as possible from each participant. Rather, students will dip in and out depending on a range of factors that are not yet fully understood.

This pilot is only a small initial step in the opening up of a vast and undeveloped field involving the reaching of youth with their permission through private messaging channels. It would be wrong to conclude from the null results here either that SMS text messaging, in general, is not a valuable channel for suicide prevention or that broad schoolwide prevention-oriented interventions cannot benefit from the addition of SMS text messaging. It could just be that our approach and the messages we used, despite the steps taken to develop them with students and to test their appeal, were not optimized for the target group. This was only a first foray into the area of broad prevention, and there are many other messaging strategies that can profitably be explored. School closures due to the COVID-19 pandemic demonstrated the need and desire to use channels of communication that extend beyond school-based activities and contact [53]. While social media channels have received a considerable amount of attention over the last decade, there remains an important role for nonpublic, nonsocial media that enables people to interact in private and safe spaces. It may well be worth examining the development of technology-enabled interventions in nonsocial channels.

While this study focused on SMS text messaging as an intervention channel, future research should explore other digital communication platforms that are popular among adolescents. These could include direct messaging on social media platforms (eg, Instagram [Meta] and Facebook [Meta]), messaging apps (eg, Snapchat [Snap Inc] and WhatsApp [Meta]), direct messages on social video platforms (eg, TikTok [ByteDance] and YouTube [Google]), or even within social gaming environments (eg, Discord and in-game chat systems). Each of these platforms has a unique communication style and user base, which could potentially increase engagement and effectiveness for specific subgroups of students. Future studies should consider including questions about preferred communication channels in the baseline assessment. This would allow researchers to tailor the intervention delivery to individual preferences, potentially increasing engagement and efficacy. Furthermore, meeting students on platforms where they already spend time could lead to a more natural integration of the intervention into their daily lives. However, it is important to note that using these platforms would require adjustments to the intervention content and delivery style to match the norms and expectations of each platform. In addition, researchers should explore innovative approaches, such as integrating mental health messaging into video gaming platforms, as mentioned earlier in our discussion about reaching less socially-connected youth. As digital communication landscapes continue to evolve rapidly, maintaining flexibility in intervention delivery channels will be crucial for future school-based suicide prevention efforts.

### Limitations

Our field test of this extension of Sources of Strength was conducted with ninth graders, targeting a particularly stressful moment in the school career of students as they moved from middle school to high school. Following the urging of the school, we rolled the program out to the whole student body for the pilot RCT, but it is possible that this was too large a leap and that the acceptability of the program to ninth graders was not replicated for other grades. The mean scores for depression and suicide ideation were very low, as one might expect from a non-clinical sample. It is possible that the program did not have any detectable effects because the floor effects were so low that it was difficult to detect overall group differences.

Several other limitations should be noted. Although samples used in pilot studies are often not entirely representative [59], the generalizability of our trial’s results is limited due to the inclusion of only 1 student body and lower-than-expected numbers of returned parental consent forms. Text4Strength was implemented in addition to a very intensive school-based suicide prevention program; the effects of the intervention may thus be depressed or underestimated. It is also possible that the program described here might have use for improving outcomes when other prevention programs are not implemented.

**Conclusions**

In contrast to a promising field test of the Text4Strength intervention conducted previously, the results of this pilot RCT suggest that the intervention is unlikely to have impacted the outcomes of interest and that undetected moderate or large effects can be ruled out with high confidence. Although motivated by the need to reach more isolated students, results showed that students with fewer friends did not engage more or show a greater effect than other participants. However, the results here are limited to a single high school already implementing an evidence-based schoolwide prevention program, setting a high bar to detect an effect beyond that of the existing program. Future interventions might benefit from exploring alternative delivery channels, such as gaming platforms or more sophisticated interactive messaging systems, and should focus on developing more personalized approaches that account for individual timing preferences and communication styles.

## Appendix

Multimedia Appendix 1 CONSORT-eHEALTH checklist (V 1.6.1).

## Abbreviations

CONSORT: Consolidated Standards of Reporting Trials
HIPAA: Health Insurance Portability and Accountability Act
PROMIS: Patient-Reported Outcomes Measurement Information System
RCT: randomized controlled trial
REDCap: Research Electronic Data Capture
